# Dietary factors and prevalence of overweight and obesity among medical students in Kancheepuram: A cross-sectional study

**DOI:** 10.6026/973206300213795

**Published:** 2025-10-31

**Authors:** Surya Senthil, Bethamcherla Bala Chandrudu, R. Naveen Shyam Sundar, Shoraf Pascal, Mohammed Zakiullah Shareef, Dinesh Raja, Balaji Arumugam

**Affiliations:** 1 Department of General Medicine, Saveetha Medical College, Saveetha Institute of Medical and Technical Sciences, Thandalam, Chennai, Tamil Nadu, India; 2 Department of Medical Physiology, Visvabharathi Medical College and General Hospital, Penchikalapadu, Kurnool Andhra Pradesh, India; 3 Department of Community Medicine, Shri Sathya Sai Medical College and Research Institute SBV Deemed to be University, Chennai, India; 4 Department of Community Medicine, Madha Medical College, Kovur, Chennai, Tamil Nadu, India; 5 Department of Internal Medicine, Satyadev Hospital, Anakapalli andhra Pradesh, India; 6 Department of Emergency Medicine, CSR Hospital, Coimbatore, Tamil Nadu, India; 7 Department of Community Medicine, Arunai Medical College and Hospital, Nassipatti, Tiruvannamalai, Tamil Nadu, India

**Keywords:** Overweight, obesity, dietary habits, medical students, fast food, cross-sectional study, body mass index (BMI), lifestyle factors

## Abstract

The prevalence of overweight and obesity and examined associated dietary factors among 138 medical students in Kancheepuram is of interest. Anthropometric measurements were recorded and dietary patterns were analyzed using a validated food frequency questionnaire. The overall prevalence of overweight and obesity was 34.1% and 11.6%, respectively. High fast-food consumption, frequent late-night eating and low fruit intake were significantly associated with elevated BMI. Thus, we show the need for targeted nutritional interventions within medical student populations.

## Background:

Globally, overweight and obesity is an important public health issue, impacting all age groups, including young adults at relatively low risk traditionally [1]. According to the World Health Organization, more than 1.9 billion adults are overweight and over 650 million of those qualify as obese, being major contributors to the global burden of non-communicable diseases [2]. Early adulthood is a significant point in the trajectory of weight gain, as excessive body fat at this time is linked to longer term risks of type 2 diabetes, cardiovascular disease, metabolic syndrome and premature death [3]. Medical students are a population of concern. Although medical students have a theoretical understanding of nutrition and health, the lifestyle that accompanies their transition to higher education is often one of irregular eating, insufficient sleep, low exercise and excessive academic stress [4]. These behavioural transitions leave medical students vulnerable to weight gain and other metabolic imbalances. In addition, medical students may understand and be aware of the importance of preventative health strategies but continue to engage in unhealthy behaviours, which can be detrimental to their personal health and to their integrity as future healthcare professionals [5]. Overweight and obesity levels among medical students in India depict broader epidemiological patterns in the country. Among young adults, the rapid urbanization, change in dietary patterns and shift to a sedentary lifestyle are contributing to rising rates of obesity [6]. Evidence reported from cross-sectional studies in major Indian cities find overweight and obesity prevalence of medical undergraduates reported between 15 and 30% [7]. However, there is a lack of evidence from semi-urban areas, where exposure to a westernized food culture occurs with traditional diets, creating a different dietary risk profile [8]. Dietary factors continue to be considered as the foundation of overweight and obesity. Reliance on calorie-dense processed foods, fried snacks and sugar-sweetened drinks and lower or no reliance on fruits, vegetables and fiber-dense foods are frequently associated with poor weight outcomes [9].

Skipping meals, especially breakfast and having inconsistent meal schedules, both appear to be common behaviors in students, are potentially more disruptive to metabolic homeostasis [10]. While the Durnin-Womersley four-site skinfold technique and some anthropometric methods are reliable and inexpensive ways to estimate percentage body fat in low-resource and low-cost settings, clinically relevant estimates of metabolic risk can be meaningful and provides insight into various other metabolic risks [11]. These risks are exacerbated by physical inactivity. Medical students are typically aware of the advantages of exercise, but compliance regarding recommended levels of activity is often lacking. Academic obligations, clinical rotations and the associated stress of exams often limit opportunities for regimented exercise [12]. Moreover, increased dependency upon electronic devices has made sedentary lifestyles more common, contributing to weight gain [13]. Being overweight and obese as a young adult may carry psychological consequences as well, such as poor body image, low self-worth and depressive symptoms [14]. Such psychosocial strains may lead to reduced motivation to engage in healthy behaviors, which may lead to an increased cycle of weight gain and psychological burden. This is also concerning in medical education as it may decrease academic performance and professional growth as well [15]. The Kancheepuram district located in Tamil Nadu is appropriate semi-urban context with local food practices that are trending toward modern dietary practice and right of Afghanistan food practices and degree of importance is increasing. Assessing medical students in this region offers insight into both the magnitude of the problem and its modifiable determinants. Therefore, it is of interest to report the prevalence of overweight and obesity among medical students in Kancheepuram and to describe the dietary determinants contributing to body weight in this population.

## Materials and Methods:

This cross-sectional study was conducted among undergraduate medical students enrolled in a private medical college in Kancheepuram district, Tamil Nadu, over a period of three months. A total of 138 students aged 18-25 years were selected using stratified random sampling, ensuring representation from all academic years. After obtaining informed consent, participants completed a self-administered, pre-validated questionnaire covering dietary habits, meal frequency, fast food consumption, fruit and vegetable intake, breakfast skipping and late-night eating patterns. Anthropometric measurements were recorded using standardized procedures. Body weight was measured using a digital scale to the nearest 0.1 kg and height was recorded using a stadiometer to the nearest 0.1 cm. Body Mass Index (BMI) was calculated as weight in kilograms divided by the square of height in meters (kg/m^2^). Overweight and obesity were classified according to WHO Asia-Pacific guidelines: BMI ≥23-24.9 kg/m^2^ as overweight and ≥25 kg/m^2^ as obese. Data were analyzed using SPSS version 26. Descriptive statistics were used to summarize participant characteristics and dietary behaviors. Chi-square test and logistic regression were applied to determine associations between dietary factors and overweight/obesity. A p-value <0.05 was considered statistically significant.

## Results:

Out of 138 medical students included in the study, 47 (34.1%) were overweight and 16 (11.6%) were obese according to WHO Asia-Pacific BMI classification. The majority of students were aged 20-22 years, with an almost equal distribution of males and females. Male students exhibited a higher prevalence of overweight and obesity compared to females. Lifestyle factors significantly influenced weight status: frequent fast-food consumption, late-night snacking and low fruit and vegetable intake were all associated with elevated BMI. Breakfast skipping was more common among overweight and obese students, suggesting irregular meal patterns contribute to weight gain. Academic year also showed a relationship with BMI, as pre-final and final year students had higher obesity prevalence, potentially reflecting lifestyle changes during advanced training. Physical activity demonstrated a protective effect, with students exercising ≥3 times per week showing lower obesity rates. High consumption of sugar-sweetened beverages was more frequent among overweight and obese students, further emphasizing the role of dietary habits in weight status. Overall, the findings highlight that both dietary patterns and lifestyle behaviors are closely linked to BMI in medical students.

Table 1 (see PDF) shows a majority of the students were in the 20-22 age group, with a nearly equal distribution of males and females. Table 2 (see PDF) shows Overall, 34.1% of students were overweight and 11.6% were obese. Table 3 (see PDF) shows Males had a higher prevalence of overweight and obesity compared to females. Table 4 (see PDF) shows Fast-food intake ≥3 times/week was significantly associated with overweight/obesity. Table 5 (see PDF) shows Breakfast skipping was more common among overweight/obese individuals. Table 6 shows Low fruit and vegetable intake (<2 servings/day) correlated with higher BMI. Table 7 (see PDF) shows Students with frequent late-night snacking had a significantly higher prevalence of elevated BMI. Table 8 (see PDF) shows among different academic years, pre-final and final year students showed higher obesity prevalence. Table 9 (see PDF) shows Students engaging in physical activity ≥3 times/week had a lower rate of obesity. Table 10 (see PDF) shows Consumption of sugar-sweetened beverages ≥4 times/week was more common among those with elevated BMI.

## Discussion:

This study reveals a concerning prevalence of overweight (34.1%) and obesity (11.6%) among medical students in Kancheepuram, underscoring the vulnerability of this population to lifestyle-related health risks. Despite their medical knowledge, students exhibited poor dietary practices such as frequent fast-food intake, late-night snacking and inadequate fruit and vegetable consumption-key contributors to elevated BMI [16]. These findings mirror global trends showing rising obesity even among healthcare students due to the pressures and lifestyle disruptions associated with medical training [17]. Male students were more likely to be overweight or obese compared to females, a trend observed in similar studies, possibly due to differences in food choices, eating frequency and physical activity. Students in senior years (final/internship) also showed higher obesity rates, likely due to increased academic stress, less time for exercise and reliance on quick, calorie-dense meals [18]. Moreover, skipping breakfast-a habit common among students-was strongly associated with higher BMI, supporting existing literature that links this behavior with metabolic dysregulation and overeating later in the day. Logistic regression analysis reinforced the strong association between dietary behaviors and weight status [19]. Fast food consumption, late-night snacking, low fruit intake and physical inactivity emerged as significant independent predictors of overweight and obesity. Notably, students who reported engaging in physical activity at least three times a week had significantly lower obesity prevalence, emphasizing the protective role of regular exercise [20]. As Students are the back bone of our future nation, healthy lifestyles and healthy food habits should be adopted from young adulthood and regular screening for overweight and obesity should be done for obesity prevention and development of complications [21]. Furthermore, these findings suggest an urgent need for structured nutrition and wellness programs within medical colleges. Targeted interventions promoting healthy eating, routine physical activity and time management can be integrated into the academic environment. An early screening and personalized counselling can play a vital role in preventing long-term health consequences in this educated yet at-risk population.

## Conclusion:

Overweight and obesity affect nearly half of the medical student population studied, driven by unhealthy dietary patterns and sedentary lifestyles. Fast food intake, late-night eating, breakfast skipping and poor fruit and vegetable consumption were significantly associated with elevated BMI. Implementing targeted health promotion strategies and lifestyle interventions within medical institutions is essential to address these modifiable risk factors and promote long-term well-being among future healthcare professionals.
